# A Simple Method to Estimate the Number of Autophagic Elements by Electron Microscopic Morphometry in Real Cellular Dimensions

**DOI:** 10.1155/2014/578698

**Published:** 2014-07-03

**Authors:** Attila L. Kovács

**Affiliations:** Department of Anatomy, Cell and Developmental Biology, Eötvös Loránd University, Budapest 1117, Hungary

## Abstract

Autophagic elements typically appear as spherical bodies. During their life they undergo a series of changes (e.g., fusion, degradation of content, and swelling) which influence their size in a way that may be characteristic for cell type, stage of maturation, or various experimentally manipulated parameters. A simple and time efficient method is suggested here to use exactly calculated specific surface values and estimate average diameter and number of autophagic elements in real cellular dimensions. The method is based on the easiest morphometric determination of relative surface (surface density) and volume (volume density) data by electron microscopy. A series of data from real experimental samples of liver and exocrine pancreatic cells are offered to illustrate the potential of these measurements and calculations.

## 1. Introduction

The most frequently applied and easiest method for electron microscopic morphometry is the so-called volumetric measurement, when we determine the volume of some components within a unit of test volume [1–3]. With regard to autophagy it means the determination of the relative volume of an autophagic compartment (*V*_*v*_) in a unit volume of cytoplasm (*V*_*c*_), reasonably expressed as *V*_*v*_ *μ*m^3^/*μ*m^3^. It is also possible and easy to determine the relative surface of an autophagic compartment (*S*_*v*_) within the same system of measurements expressed as *S*_*v*_ *μ*m^2^/*μ*m^3^ [1, 2]. In addition to traditional point counting, computer aided techniques are also available for this purpose (e.g., NIH ImageJ and Adobe Photoshop).

The values that we can get by these morphometric measurements include the total size of both the surface (*S*_*v*_) and the volume (*V*_*v*_) of the autophagic elements in 1 *μ*m^3^ of cytoplasm, also named, respectively, as surface and volume density. *V*_*v*_ data in articles are usually given as multiplied with 100 to show the % volume of the autophagic compartment within the cytoplasm.

**Figure 1:**
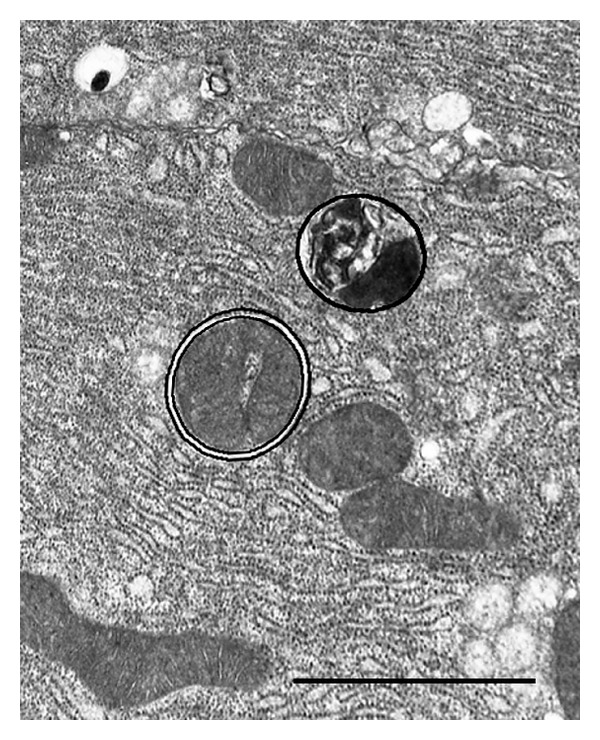
An electron microscopic picture showing a portion of a pancreatic acinar cell with an autophagosome encircled by double line and an autolysosome, encircled by single line, along their bordering membranes. Autophagosomes and autolysosomes are usually taken as two different categories of autophagic vacuoles. For *V*_*v*_ data we measure the area within the membrane and for *S*_*v*_ data the length of the bordering membrane separating the autophagocytosed material from the cytosol. These data are then related to the size of the surrounding cytoplasmic area during simple morphometric calculations. Scale bar 1 *μ*m.

The theoretical background and the practical description of *S*_*v*_ and *V*_*v*_ measurements are beyond the scope of the present paper; however, they are available in a well-illustrated manner in several reviews and books like [1–3]. In short, for the determination of *V*_*v*_ we measure areas and for *S*_*v*_ length of limiting membranes of organelles on images from electron microscopic sections, as basic data for subsequent calculations (Figure 1).

While *V*_*v*_ is good to express the total volume of the autophagic compartment in a given volume of cytoplasm, it does not provide any information about the number of autophagic elements behind it. In many cases it would be very useful to estimate at least the approximate number of various autophagic components. These types of data could help to develop, in real cellular dimensions, better understanding of qualities of autophagy which depend on the number of objects.

Methods to determine the exact number of components in a given volume (numerical density) have been worked out and are described in detail both in the older literature (based on the analysis of profile histograms [2]) and in the new era of morphometry using the disector technique [3]. However, autophagy is a special object for morphometry as autophagic elements usually comprise a rather small proportion of the cytoplasmic volume; therefore, a very large test area must be evaluated for the measurements by the histograms and the dissector technique. The application of these accurate methods for autophagy studies would, therefore, be too time consuming and tedious for routine use. That is the most likely reason why, to my knowledge, such publications have not appeared so far. Here I suggest a simple and efficient approach which utilizes specific surface values (*S*_*sp*_ = *S*_*v*_/*V*_*v*_ *μ*m^2^/*μ*m^3^) for the determination of the number of autophagic elements. To illustrate its potentials I apply it to a large set of data from my previous measurements on liver and exocrine pancreatic cells with variable autophagic activity. The presented method offers approximations with a reasonable bias and can be utilized for a quick and low effort characterization of autophagy by the above parameters. The rough estimations obtained by this approach may also help to select specific cases to be evaluated by the accurate, high investment histogram and disector methods.

## 2. Results and Discussion

*S*_*sp*_ gives us the surface of the (autophagic) compartment that belongs to a unit volume of the same (autophagic) compartment. The fact that the autophagic elements have the geometry of a sphere as a rule gives us the possibility to estimate their number with the help of their *S*_*sp*_. The method of estimation is based on the simple geometrical fact that the surface/volume ratio of a certain sphere is exactly determined by its size. Therefore, for a homogenous population of spheres it is possible to calculate the diameter from their *S*_*sp*_ value on the base of the following formulas:

(1) $$\begin{array}{c} S=D^{2}\pi ;\;V=\frac{D^{3}\pi }{6};\;S_{sp}=\frac{S}{V};\;D=\frac{6}{S_{sp}}, \end{array}$$

where *S*, *V*, and *D* are the surface, volume, and diameter of a sphere, respectively.

To illustrate the technique for the determination of numbers of spheres, let us suppose that we have a homogenous population of spherical bodies in the cytoplasm. We measure *S*_*v*_ and *V*_*v*_ by simple morphometry and compute *S*_*sp*_(*S*_*v*_/*V*_*v*_) with them. With the diameter (*D*_*c*_) calculated from *S*_*sp*_(*D*_*c*_ = 6/*S*_*sp*_), we can also calculate the individual volume of a single sphere in this homogenous population (*V*_*c*_ = *D*_*c*_^3^ *π*/6). As we have the total of individual volumes (*V*_*v*_), with a single division we can get the precise number of spheres in the unit volume containing those spheres. For example, if in a homogenous population we measure an *S*_*v*_ and *V*_*v*_ of 0,0351 *μ*m^2^/*μ*m^3^ and 0,0041 *μ*m^3^/*μ*m^3^_,_ respectively, the *S*_*sp*_ from them will be 8,55 *μ*m^−1^. This in turn gives a diameter of 0,7 *μ*m with the help of the formula *D*_*c*_ = 6/*S*_*sp*_. The volume of a sphere with a diameter of 0,7 *μ*m is 0,180 *μ*m^3^. The total volume of the population of spheres in a cell with 5000 *μ*m^3^ size (the approximate average size of a rat hepatocyte [4]) will be 5000 × 0,0041 = 20,5 *μ*m^3^. A single division of the total by the individual volumes (20,5/0,180) will give us the number, which is 114 in this case. This calculation, as mentioned above, gives the precise number for a uniform population of spheres with equal diameter.

However, the autophagic elements have variable size which causes a bias in estimating numbers with the presented method. To estimate this bias we can make model calculations with sets of data in the range of real life samples. Although it is only an approximation, for our purpose it is possible to consider the distribution of the diameter values as closely Gaussian.

The typical diameter range for autophagosomes in mammalian cells, according to my own unpublished measurements and data derived from various articles [5–9], usually falls within 0,7–1,1 *μ*m. Taking data in this range we can calculate the real average volume and the volume estimated with the help of the diameter derived from the *S*_*sp*_ value based on the *D*_*c*_ = 6/*S*_*sp*_ formula (calculations are presented in the Supplementary Material available online at http://dx.doi.org/10.1155/2014/578698). The result of this probe shows an underestimation of the number by a factor of 1,06. Further analysis reveals that the error of estimation depends on the changes in size distribution. Stimulation of autophagy, transition of autophagosomes to autolysosomes, and the following fusion events result in the widening of the distribution together with an appearance of categories with bigger size. The analysis of a probe with a range of 0,7–1,4 *μ*m diameter, and a tail at the right end of the distribution, results in a 15,54% underestimation of the number (see the details of the calculations in the Supplementary Material).

Volumetric analysis of autophagy by point counting electron microscopic morphometry works with rather high standard errors [10–18], and sometimes only relatively big changes can be found statistically significant. Results presented here show that although the calculation of numbers of autophagic elements from *S*_*sp*_ leads to underestimation, the error remains within a rather narrow range. In addition, if considered necessary, they might even be corrected with the help of size distribution data (see Supplementary Material). Being a sensitive indicator of changes related to the size of autophagic elements, *S*_*sp*_ is a valuable parameter in itself. The derived *D*_*c*_ and *N*_*c*_ values also express quantitative changes with reasonably good approximation. In addition they help to depict the events during autophagy in real cellular dimensions.

In the following section I illustrate the utilization of this approach in selected autophagic processes. Some of them were previously described by volumetric (*V*_*v*_) evaluation. For the present purpose a review and additional measurements were made to expand our database and support the calculation of *S*_*sp*_.

Table 1 shows that *S*_*sp*_ data are characteristically different for various categories of the autophagic-lysosomal compartment. The effect of various experimental treatments is also reflected in their values. The *S*_*sp*_ is highest (9,9; 11,0) in the case of typical dense bodies resulting in small *D*_*c*_ (0,61; 0,55 *μ*m). A rather wide distribution of *S*_*sp*_ data for autolysosomes is revealed in different experimental systems and treatments. Low *S*_*sp*_ values correlate well with swelling in 3 h amino acid (5,1) or propylamine treatment (2,5) and extensive fusion in leupeptin treatment (3,9). These features are only qualitatively indicated by the simple morphological evaluation of the pictures. It is the population of autophagosomes which appears to be the least heterogeneous. *S*_*sp*_ values for autophagosomes are 6,5–8,5 which correspond to a *D*_*c*_ range of 0,71–0,92 *μ*m.

The last column of Table 1 shows the calculated number of autophagic structures in a real cellular volume of 5000 *μ*m^3^, an average rat liver hepatocyte [4]. For better comparison and simplicity, I have chosen the same volume for the exocrine pancreas cells.

The calculated numbers (*N*_*c*_) are especially valuable to give a graphic quantitative picture of the autophagic lysosomal compartment in real cellular dimensions. The total number of autophagic elements may span a range of four orders of magnitude (1–1000) in a cell depending on experimental conditions. Immediately after feeding or amino acid treatment, when autophagy and lysosomal protein degradation are minimal, the number of autophagosomes may remain under or close to 10. Autolysosomes, however, are present in the lower range and dense bodies in the middle range of the 10^1^ order of magnitude. Fasting for 24 h in vivo increases the number of autophagosomes several times in liver cells. Nevertheless, the sum of autophagosomes and autolysosomes remains under 100. The number of dense bodies remains similar, although their size becomes bigger after fasting. Total amino acid withdrawal in vitro elevates the autophagosome number over 100 and that of autolysosomes close to 200.

The analysis of vinblastine treatments further illustrates the potential of the *S*_*sp*_ values in approximate calculations of component numbers. This alkaloid disrupts microtubules and inhibits fusion of autophagosomes with endosomes and lysosomes [15, 19, 20]; in addition it also stimulates autophagosome formation [18, 19, 21]. In exocrine pancreatic cells after vinblastine treatment in vivo, we observed both the highest rate of accumulation and the total volume of the autophagic compartment [21]. After a review and additional measurements I calculated with the *S*_*sp*_ method the component numbers of the autophagic compartment for certain time points.

The highest accumulation rate is seen between 1 and 1,5 h of vinblastine treatment while the highest volume at 6 h. The increase of the number of autophagic elements between 1 and 1,5 h is 148. This is the minimum number of autophagosomes generated during this 30 min. Supposing that each autophagosome is created from a single initiation event, we can calculate that the approximate frequency of initiations is 12 seconds in this case.

A calculation from the *S*_*sp*_ values at the maximal volume of the autophagic compartment, 6 h after vinblastine treatment, shows that the number of autophagic elements can exceed 1000 in exocrine pancreatic cells.

The increasing interest in autophagy research goes along with the need to apply complex methodological approaches. In spite of many new possibilities [20, 22], electron microscopy remains an option and in some cases may prove to be indispensable. The *S*_*sp*_ method might be a good and simple choice for solving problems where approximation of changes in size distribution and number is necessary.

## Supplementary Material

The supplementary material contains model calculations to illustrate the method for the estimation of the number of autophagic elements in cells. Examples for the correction of inherent bias are also included in three model distributions.

## Figures and Tables

**Table 1:** Approximate diameter (*D*_*c*_), average volume (*V*_c_), and number of autophagic elements (*N*_c_) in a cell with 5000 *μ*m^3^ cytoplasm, calculated from the specific surface values (*S*_v_ /*V*_v_ = *S*_*sp*_) from real experimental samples of liver and exocrine pancreatic cells. The details of calculation are described in the text.

Experimental system and treatment	Category	*S*_v_ *μ*m^2^/*μ*m^3^	*V*_v_ *μ*m^3^/*μ*m^3^	*S*_*sp*_ *μ*m^2^/*μ*m^3^	*D*_c_ *μ*m	*V*_c_ *μ*m^3^	*N*_c_ in 5000 *μ*m^3^
Isolated hepatocytes							
Plus amino acid mixture 30 min	Afs	0,0023	0,0003	7,6	0,79	0,26	6
	Al	0,0147	0,0022	6,8	0,88	0,36	30
Plus amino acid mixture 3 h	Afs	0,0047	0,0006	7,4	0,81	0,28	11
	Al	0,0367	0,0072	5,1	1,18	0,85	42
Minus amino acid mixture 3 h 30 min	Afs	0,0472	0,0067	7,0	0,86	0,33	102
	Al	0,1333	0,0215	5,9	0,97	0,47	195
Propylamine 10 mM 3 h	Afs	0,0856	0,0124	6,9	0,87	0,34	180
	Al	0,0563	0,0084	6,7	0,90	0,38	112
	Alam	0,2925	0,1170	2,5	2,40	7,24	81

Hepatocytes in vivo							
3 h feeding	Afs	0,0024	0,0003	7,9	0,76	0,23	7
	Al	0,0040	0,0005	7,9	0,76	0,23	11
	Db	0,0088	0,0008	11,0	0,55	0,08	47
24 h fasting	Afs	0,0101	0,0014	7,2	0,84	0,31	23
	Al	0,0171	0,0022	7,8	0,77	0,24	46
	Db	0,0079	0,0008	9,9	0,61	0,12	34
Ad libitum feeding	Afs	0,0025	0,0003	8,5	0,71	0,18	8
	Al	0,0048	0,0006	8,0	0,75	0,22	14
Vinblastine treatment 0,1 mg/g 2 h	Afs	0,0778	0,0105	7,4	0,81	0,28	189
	Al	0,0648	0,0086	7,5	0,80	0,26	163
Leupeptin treatment 0,12 mg/g 2 h	Afs	0,0450	0,0054	8,3	0,72	0,20	138
	Al	0,1095	0,0278	3,9	1,52	1,85	75

Exocrine pancreas cells in vivo							
24 h fasting	Afs	0,0050	0,0007	7,2	0,83	0,30	12
	Al	0,0061	0,0009	6,8	0,89	0,36	12
Ad libitum feeding	Afs	0,0016	0,0002	7,9	0,76	0,23	4
	Al	0,0024	0,0003	7,8	0,77	0,23	6
Vinblastine treatment 0,1 mg/g 1 h	Afs	0,0447	0,0063	7,1	0,85	0,32	99
	Al	0,0233	0,0034	6,8	0,88	0,35	48
Vinblastine treatment 0,1 mg/g 1,5 h	Afs	0,1073	0,0159	6,8	0,89	0,37	216
	Al	0,0401	0,0060	6,7	0,90	0,38	79
Vinblastine treatment 0,1 mg/g 6 h	Afs	0,3362	0,0518	6,5	0,92	0,41	626
	Al	0,3081	0,0517	6,0	1,01	0,53	484

Afs: autophagosome (early autophagic vacuole), Al: autolysosome (late autophagic vacuole), Db: dense body, Alam: swollen electron-lucent amine type of autolysosome.

The experimental animals were from male mice for in vivo treatments and from rats for isolated cells. The evaluated cytoplasmic area was in the range of 9–16000 *μ*m^2^. For further details of electron microscopy, morphometry, and experimental systems see, for example, in [5–10].
