# Supplementary material for: A Simple Method to Estimate the Number of Autophagic Elements by Electron Microscopic Morphometry in Real Cellular Dimensions
Source: Biomed Res Int. 2014 Jul 3;2014:578698. doi: 10.1155/2014/578698 (PMC4106081; doi:10.1155/2014/578698)
Supplement: Supplementary file 1 — The supplementary material contains model calculations to illustrate the method for the estimation of the number of autophagic elements in cells. Examples for the correction of inherent bias are also included in three model distributions. [file 578698.f1.docx]

**Supplementary calculations for the estimation of the number of autophagic elements in real cellular dimensions**

Measurements on experimental samples we get Sv and Vv values which correspond to the sum of S and V data in our model samples below. By Sv (surface of objects/cytoplasmic volume) and Vv (volume of objects/cytoplasmic volume) we calculate Ssp both here and in real experiments. Using Ssp we in turn can calculate the estimated diameter (Dc) and volume (Vc) for the estimation of number.

The course of calculations is the following. A set of data is given for the model calculations which cover a size range of spheres expressed as D (Table 1 suppl.). First Dc (estimated diameter) is calculated by the formula 6/Ssp. With the help of Dc the estimated volume of a single sphere (Vc) can be derived. By the ratio of Vv/Vc we can calculate the estimated number. The ratio of the known and calculated number gives us the estimation of bias.

A set of spheres with known number and approximate Gaussian distributions (normal (Table 2 suppl.), narrow (Table 3 suppl.), and with a tail in the higher region (Table 4 suppl.) is given below to roughly estimate the extent and variability of the inherent bias.

Table 1 suppl.

Database for the model calculations: surface (S) amd volume (V) values for a set of spheres with the given diameters (D)

|  | D; (μm) | S; (D^2^π μm^2^) | V; (D^3^π/6μm^3^) |  |
| --- | --- | --- | --- | --- |
|  | 0,6 | 1,130973355 | 0,113097336 |  |
|  | 0,7 | 1,5393804 | 0,17959438 |  |
|  | 0,8 | 2,010619298 | 0,268082573 |  |
|  | 0,9 | 2,544690049 | 0,381703507 |  |
|  | 1 | 3,141592654 | 0,523598776 |  |
|  | 1,1 | 3,801327111 | 0,69690997 |  |
|  | 1,2 | 4,523893421 | 0,904778684 |  |
|  | 1,3 | 5,309291585 | 1,15034651 |  |

Table 2 suppl.

Spheres with normal distribution

| Real values | | | | |
| --- | --- | --- | --- | --- |
| Number | D | S | V | Sv/Vv (Ssp) |
| 1 | 0,6 | 1,130973355 | 0,113097336 |  |
| 2 | 0,7 | 1,5393804 | 0,17959438 |  |
| 3 | 0,7 | 1,5393804 | 0,17959438 |  |
| 4 | 0,8 | 2,010619298 | 0,268082573 |  |
| 5 | 0,8 | 2,010619298 | 0,268082573 |  |
| 6 | 0,8 | 2,010619298 | 0,268082573 |  |
| 7 | 0,9 | 2,544690049 | 0,381703507 |  |
| 8 | 0,9 | 2,544690049 | 0,381703507 |  |
| 9 | 1 | 3,141592654 | 0,523598776 |  |
|  |  |  |  |  |
| Sum of S (Sv μm^2^/ μm^3^) and V (Vv μm^3^/ μm^3^); Ssp (μm^2^/ μm^3^) |  | 18,4725648 | 2,563539605 | 7,205882 |
|  |  |  |  |  |
| Dc calculated from Ssp (6/Ssp) | 0,832653061 |  |  |  |
| Vc from calculated Dc (D^3^π /6) | 0,302267095 |  |  |  |
| Real number | 9 |  |  |  |
| Calculated number (Vv/Vc) | 8,481040946 |  |  |  |
| Precision of estimation (%) | -5,766211713 |  |  |  |
| Correction factor | 1,06119049 |  |  |  |

Table 3 suppl.

Spheres with narrow distribution

| Real values | | | | |
| --- | --- | --- | --- | --- |
|  | | | | |
| Number | D | S | V | Sv/Vv (Ssp) |
| 1 | 0,6 | 1,130973355 | 0,113097336 |  |
| 2 | 0,7 | 1,5393804 | 0,17959438 |  |
| 3 | 0,8 | 2,010619298 | 0,268082573 |  |
| 4 | 0,8 | 2,010619298 | 0,268082573 |  |
| 5 | 0,8 | 2,010619298 | 0,268082573 |  |
| 6 | 0,8 | 2,010619298 | 0,268082573 |  |
| 7 | 0,8 | 2,010619298 | 0,268082573 |  |
| 8 | 0,9 | 2,544690049 | 0,381703507 |  |
| 9 | 1 | 3,141592654 | 0,523598776 |  |
|  |  |  |  |  |
| Sum of S (Sv μm^2^/ μm^3^) and V (Vv μm^3^/ μm^3^);  Ssp (μm^2^/ μm^3^) |  | 18,40973295 | 2,538406864 | 7,252475 |
|  |  |  |  |  |
| Dc calculated from Ssp (6/Ssp) | 0,827303754 |  |  |  |
| Vc from calculated Dc (D^3^π /6) | 0,296478775 |  |  |  |
| Real number | 9 |  |  |  |
| Calculated number (Vv/Vc) | 8,561850213 |  |  |  |
| Precision of estimation % | -4,868330961 |  |  |  |
| Correction factor | 1,051174662 |  |  |  |

Table 4 suppl.

A given set of spheres with wider distribution with a tail in the higher region

| Real values | | | | |
| --- | --- | --- | --- | --- |
| Number | D | S | V | Sv/Vv (Ssp) |
| 1 | 0,6 | 1,130973355 | 0,113097336 |  |
| 2 | 0,6 | 1,130973355 | 0,113097336 |  |
| 3 | 0,7 | 1,5393804 | 0,17959438 |  |
| 4 | 0,7 | 1,5393804 | 0,17959438 |  |
| 5 | 0,7 | 1,5393804 | 0,17959438 |  |
| 6 | 0,8 | 2,010619298 | 0,268082573 |  |
| 7 | 0,8 | 2,010619298 | 0,268082573 |  |
| 8 | 0,8 | 2,010619298 | 0,268082573 |  |
| 9 | 0,9 | 2,544690049 | 0,381703507 |  |
| 10 | 0,9 | 2,544690049 | 0,381703507 |  |
| 11 | 1 | 3,141592654 | 0,523598776 |  |
| 12 | 1,1 | 3,801327111 | 0,69690997 |  |
| 13 | 1,2 | 4,523893421 | 0,904778684 |  |
| 14 | 1,3 | 5,309291585 | 1,15034651 |  |
| Sum of S (Sv μm^2^/ μm^3^) and V (Vv μm^3^/ μm^3^);  Ssp (μm^2^/ μm^3^) |  | 34,77743068 | 5,608266485 | 6,201102 |
|  |  |  |  |  |
| Dc calculated from ssp (6/Ssp) | 0,967570009 |  |  |  |
| Vc from calculated Dc (D^3^π /6) | 0,47429202 |  |  |  |
| Real number | 14 |  |  |  |
| Calculated number | 11,82450105 |  |  |  |
| Precision of estimation % | -15,53927825 |  |  |  |
| Correction factor | 1,183982305 |  |  |  |

The above calculations show that distribution patterns influence the precision of estimation in such a way that widening size distribution increases bias. Although it is not exact in pure mathematical sense, this bias can be approximated in real experimental samples by making model calculations based on profile diameter distributions as illustrated above by the model samples.

Various types of autophagic vacuoles show different size distributions usually with autophagosomes being most and late autolysosomes least uniform respectively. Accordingly, the precision of estimation by the presented method also varies for these different categories. As changes in the number of autophagic vacuoles in various conditions may cover one or even more orders of magnitude, the bias even in the widest distribution of sizes will give meaningful results for most of the cases.
